# Reconstructive Therapy of an Adenomatoid Odontogenic Tumor Associated with an Erupted Maxillary Lateral Incisor: Report of a Rare Case: a Rare Case in Reconstructive Therapy of an Adenomatoid Odontogenic Tumor

**DOI:** 10.31661/gmj.v13iSP1.3643

**Published:** 2024-12-09

**Authors:** Sahar Chokami Rafiei, Zahra Jafary Nodoushan

**Affiliations:** ^1^ Department of Periodontology, Research Center for Prevention of Oral and Dental Diseases, Baqiyatallah University of Medical Sciences, Tehran, Iran; ^2^ Department of Periodontology, School of Dentistry, Baqiyatallah University of Medical Sciences, Tehran, Iran

**Keywords:** Adenomatoid Odontogenic Tumor, Connective Tissue, Maxillary, Pedicle, Reconstructive, Therapy, Case Report

## Abstract

**Background:**

Adenomatoid odontogenic tumor is an asymptomatic, rare, benign neoplasm of odontogenic epithelium origin with a slow growth rate.

**Case Presentation:**

We describe a case of a 15-year-old male patient of Asian descent, who presented with a 0.8*0.8*0.3-cm expansile mass in the lateral incisor region, which is an uncommon location and gender for AOT. Cone-beam computed tomography revealed evidence of a well-defined unilocular radiolucent lesion in the anterior maxilla, with snowflake calcification, expansion of the buccal cortical plate, and lingual drift of the lateral incisor. After complete excision of the mass under local anesthesia and extraction of the associated tooth, the defect was filled with xenograft, covered with pedicle connective tissue graft from the palate, and sutured. Histological sections showed a benign odontogenic tumor composed of a proliferation of spindle-shaped epithelial cells forming strands and whorls structure. A thick capsule surrounded the lesion. Some basophilic calcification with concentric structures was also seen. A piece of oral mucosa covered by parakeratinized stratified squamous epithelium was evident. There was no evidence of malignancy.

**Conclusion:**

Clinical, radiographic, and histopathologic findings aided the final diagnosis of the AOT. The patient was treated surgically and later rehabilitated with a removable prosthesis. No recurrence was detected over the one-year follow-up. This study can help diagnose and manage this lesion according to its specific characteristics.

## Introduction

According to the second edition of the WHO classification, an adenomatoid odontogenic tumor (AOT) is defined as an odontogenic tumor of the epithelium with duct-like structures and varying degrees of induced changes in the connective tissue [1]. AOT is defined as a two-thirds tumor because two-thirds of cases occur in the maxilla, two-thirds occur in young women, two-thirds are related to impacted teeth, and two-thirds are related to canine teeth [2]. AOT is a single rare neoplasm with a prevalence of 1.2-7.2% among all odontogenic tumors. This tumor accounts for 1.2% of all odontogenic tumors in the Caucasian race and up to 9% of all odontogenic tumors in African ancestry. The three clinical and histopathological subtypes of AOT include follicular, extrafollicular, and peripheral. Follicular and extrafollicular types, which are intraosseous tumors, account for 97.7%, and the rare peripheral type accounts for only 2.3% of all AOT cases [3][4][5][6][7].

The follicular type is related to an unerupted tooth and is observed as a lacunar ionic radiolucency with a defined boundary in relation to the crown or root of the unerupted tooth in the graphical view that mimics the appearance of a dentigorous cyst [5]. In this article, we aim to report an unusual case of AOT and discuss the clinical, radiographic, histopathological, and therapeutic features of this case.a

## Case Description

**Figure-1:**
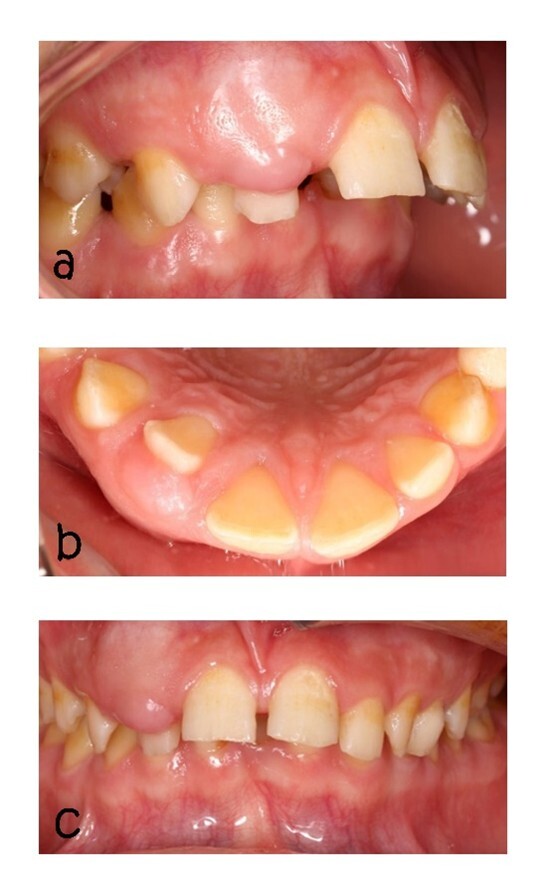
Figure 1. a: Intraoral photograph (lateral view), b: Intraoral photograph occlusal view showing shift lateral incisor palatal aspect ,c: Intraoral photograph with obliteration vestibular

**Figure-2:**
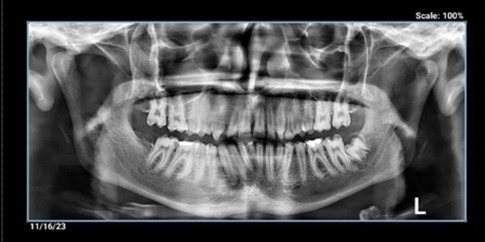
Figure 2. OPG showing a round oval radiolucency around UR2 root

**Figure-3:**
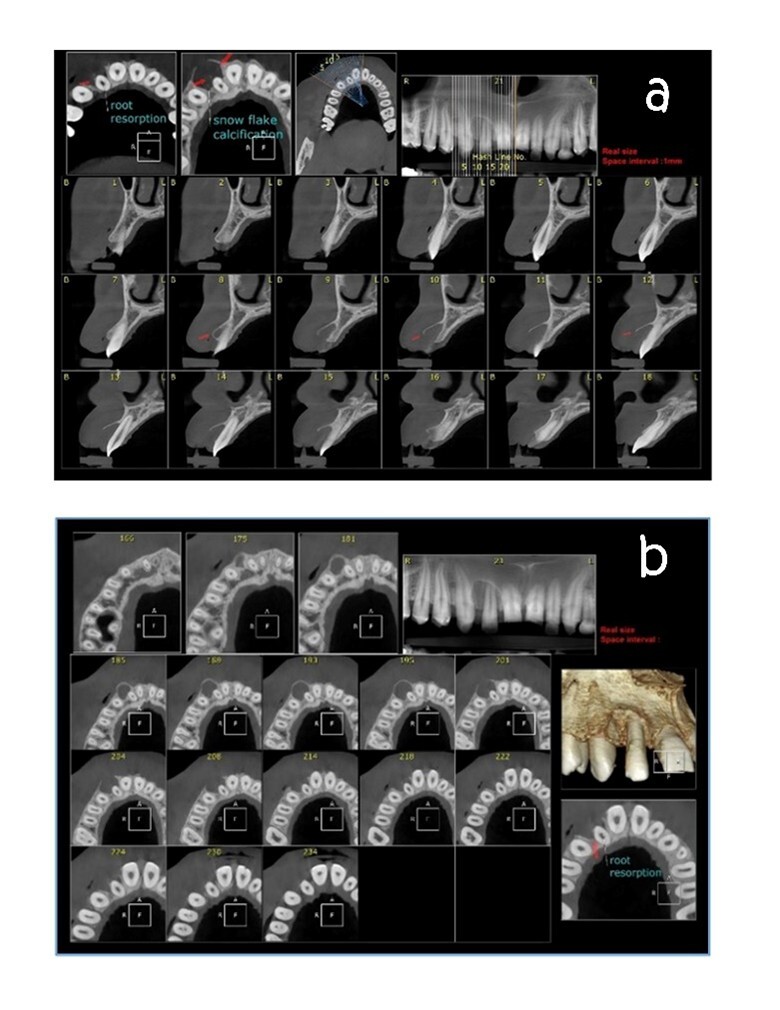
Figure 3. a: CBCT axial view showing root resorption and snow flake calcification, b: CBCT cross sectional view showing bone expantion and root resorption

We describe a case of a 15-year-old boy of Asian descent, who had a lump in the lateral maxillary incisor region with dimensions of approximately 0.3*0.8*0.8*0.3 cm, which is unusual for AOT cases in terms of the position of the affected tooth and the sex of the person involved (Figure-1). Maxillary lateral incisor tooth drift was seen (Figure-1). The mucosa above the mass was normal and did not cause pain, paresthesia, or infection. The lymph nodes were not palpable. The patient and his family members did not report a history of systemic medical problems. The patient had no history of trauma, and no asymmetry was observed in the extraoral view. The patient’s occlusion was based on Angel classification, class 2, subgroup 2 (Figure-1).

Clinical examinations showed primary canine on the left side of the maxilla and primary lateral teeth in the mandible. Periapical and panoramic radiography (Figure-2) and cone-beam computed tomography (CBCT) were taken. The graphic view consisted of a unilocular radiolucency with a well-defined boundary in the anterior maxilla, with some calcification. External root resorption was also observed in the related tooth, with buccal cortical plate extension (Figures-3a,3b).

**Figure-4:**
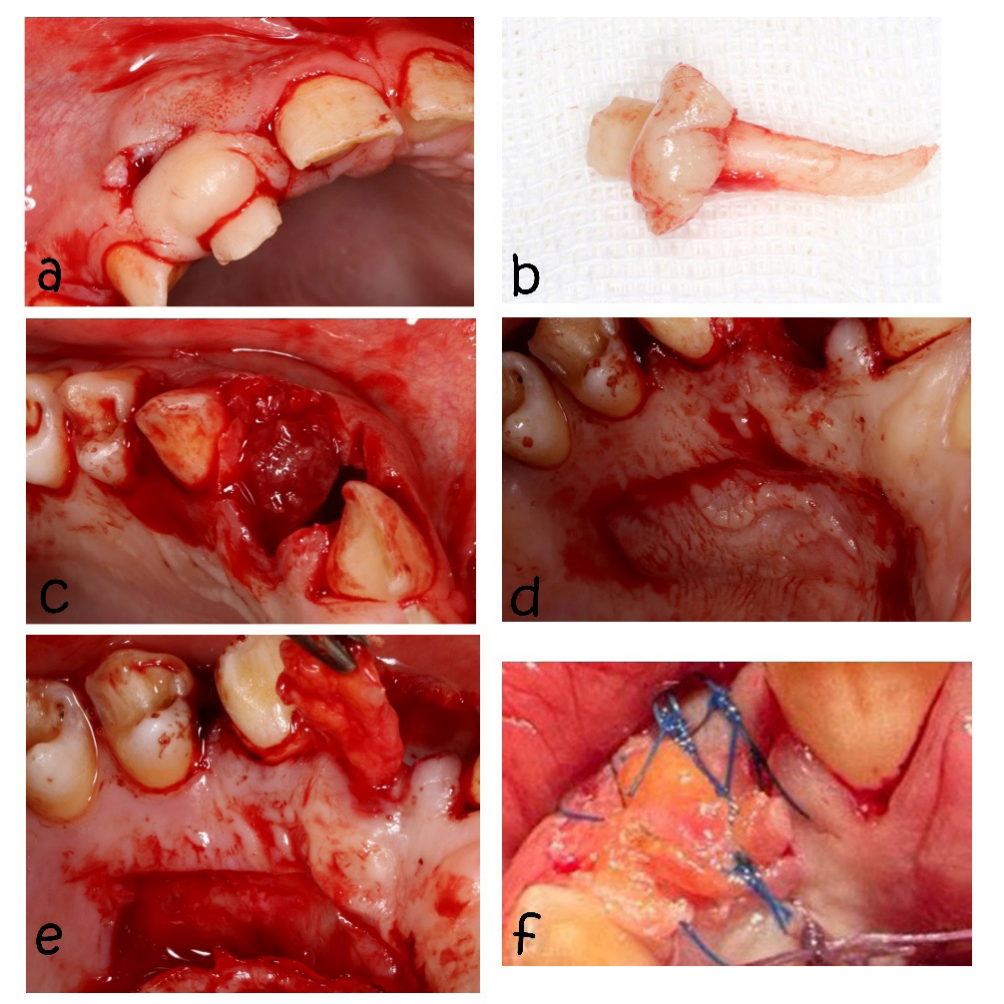
Figure 4. a:submarginal incision, b: extraction teeth, c:view of the socket of extracted tooth, d: trap door incision in the palate, e: the pedicle graft was dissected at the coronal, distal, and apical aspects, leaving the mesial side attached, f: suturing

After complete removal of the mass and extraction of the related tooth under local anesthesia (Figures-4), the lesion was filled with xenograft and sutured using the pedicle-connective tissue graft with palatal tunneling [7]. The dimensions of the socket opening were measured, and a trap door incision was performed in the palatal region of the lateral and canine teeth (Figure-4d). In the palatal side of the orifice of the socket, a tunnel was created to the receiver site. Then, the CTG was separated from the distal, upper, and lower parts, and only the mesial part remained connected; the CTG was removed from the tunnel and placed on the socket at least 3 mm under the facial tissue and sutured (Figure-4f).

Aspiration of the lesion was negative. The specimen, including an irregular piece of brown elastic tissue in formalin, was sent to the Pathology Department for histopathological examination.

**Figure-5:**
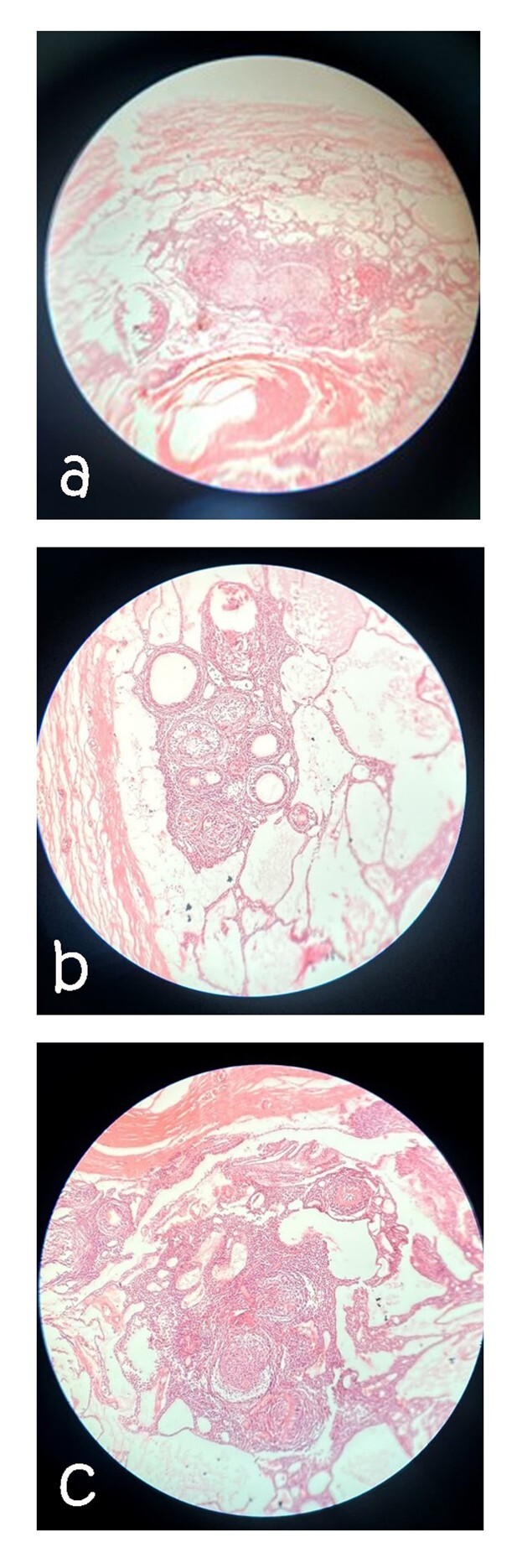
Figure 5. a: Histopathologic sections with hematoxylin-eosin stained demonstrating a tumor with a thickcapsule. (H and E × 4), b: Duct-like spaces that be polarized away from central space are surrounded by cuboidal to columnar cells. (HE × 10), c: irregular calcified areas with eosinophilic material.(H and E × 10)

Histopathological evaluation revealed an odontogenic tumor that was surrounded by a thick and fibrous capsule. Such well-defined tumors are solid with cystic formation in some case (Figure-5a). This tumor comprises spindle-shaped epithelial cells that form sheets, syncytial arrangement stands, and whorled masses of cells in a fibrous stroma(Figure-5b). Some duct-like and tubular structures surrounded by a layer of cuboidal to columnar epithelial cells are also seen in the stroma(Figure-5c). Several basophilic calcifications with concentric structures were observed.

The final diagnosis was based on clinical, radiographic, and histopathologic findings of AOT. The patient was assured that his name would not be published in the article and that his identity would remain confidential.

## Discussion

Common non-neoplastic causes of jaw swelling in young people include apical cysts, calcifying odontogenic cysts, dentigerous cysts, odontogenic keratocysts, and central giant cell granuloma. Common causes of neoplastic jaw swelling in young people include AOT, unicystic ameloblastoma, calcifying epithelial odontogenic tumors, fibromas ameloblastoma, and fibroblastoid ameloblastoma [8]. Based on clinical, radiographic, and histological findings, the final diagnosis of the present patient was AOT.

In this case, due to the association of the lesion with a protruding permanent tooth (permanent lateral incisor maxillary tooth), the type of AOT was extrafollicular (permanent incisor maxillary tooth). The peripheral type of AOT affects the incisor tooth in 90% of cases [9].

Therefore, it is unusual because our case is an extrafollicular AOT that involves the lateral tooth. The two-dimensional graphical view of the extrafollicular type resembles a periapical cyst or a periradicular cyst [10] and intrabony periodontal lesions [11]. The presence of a vital tooth associated with the lesion precludes differential diagnoses of periapical cysts or periradicular cysts, and the absence of periodontal pockets and crestal bone resorption excludes the differential diagnosis of intrabony lesions. Since the findings of the 2D radiography were not diagnostic, the patient underwent a CBCT examination, which led to the diagnosis of radiopaque centers and cortical bone extension on the X-ray film.

In 77% of AOT cases, small opacities are associated with cortical bone expansion [12].

In addition, 3D graphs will determine the tumor’s shape, spatial details of the tumor in relation to adjacent structures, severity, and three-dimensional extension of the lesion. However, in panoramic views, radiopaque centers have been seen in 50% of AOT cases [13][14].

The radiopaque view of radiolucent lesions in AOT can mimic the graphical view of calcifying odontogenic cysts and tumors [15][16].

Based on the results of a review of 272 cases with AOT diagnosis, the age range of the patients at the time of diagnosis of AOT was between 3 and 82 years (mean = 18.4 years) [12], and our patient was in the same age range. Due to the geographical aspects of the sex distribution of patients with AOT, AOT in the Asian breed tends to be more prevalent in females than males [16][17], so the gender of our case was one of the unusual cases of this case.

The treatment of choice for this tumor is enucleation and curettage [18][19]. Conservative surgical excision is usually performed because the tumor is well-encapsulated and easily separates from the surrounding bone [20].

The recurrence of this tumor is very low (approximately 0.2%) [18][19][20][21][22][23]. In the present case, the lesion was excised completely. In addition, the extraction of the affected tooth was performed based on the recommendations of the articles [24][25] and also based on the suggestions of the articles in AOT-induced intrabony defect, guided bone regeneration (GBR) was performed [8]. According to previous study, palatal pedicle graft was used for socket preservation, which improves blood vessel sources and stability compared to free graft methods and has minimal complications. According to previous studies, this method improves the buccolingual and mesiodistal dimensions over time [7]. There was no evidence of recurrence in the one-year follow-up of the patient.

## Conclusion

Increased gingival volume may indicate a malignant condition in a young person. Therefore, a thorough clinical and radiographic examination is necessary for any increase in gingival volume. In this case, immediate diagnosis and treatment reduced the patient’s complications.

## Conflict of Interest

The authors have no conflicts of interest to declare.
